# Mobile Location in Wireless Sensor Networks Based on Multi Spot Measurements Model

**DOI:** 10.3390/s22239559

**Published:** 2022-12-06

**Authors:** Chao Zheng, Wei Hu, Jiyan Huang, Pengfei Wang, Yufei Liu, Chenyu Yang

**Affiliations:** 1 Sichuan Jiuzhou Aerocont Technologies Co., Ltd., Mianyang 621000, China; 2 School of Information and Communication Engineering, University of Electronic Science and Technology of China, Chengdu 611731, China

**Keywords:** Cramer–Rao lower bound (CRLB), multi-spot measurements model, sensor localization, time of arrival (TOA)

## Abstract

The localization of sensors in wireless sensor networks has recently gained considerable attention. The existing location methods are based on a one-spot measurement model. It is difficult to further improve the positioning accuracy of existing location methods based on single-spot measurements. This paper proposes two location methods based on multi-spot measurements to reduce location errors. Because the multi-spot measurements model has more measurement equations than the single-spot measurements model, the proposed methods provide better performance than the traditional location methods using one-spot measurement in terms of the root mean square error (RMSE) and Cramer–Rao lower bound (CRLB). Both closed-form and iterative algorithms are proposed in this paper. The former performs suboptimally with less computational burden, whereas the latter has the highest positioning accuracy in attaining the CRLB. Moreover, a novel CRLB for the proposed multi-spot measurements model is also derived in this paper. A theoretical proof shows that the traditional CRLB in the case of single-spot measurements performs worse than the proposed CRLB in the case of multi-spot measurements. The simulation results show that the proposed methods have a lower RMSE than the traditional location methods.

## 1. Introduction

Wireless sensor networks (WSNs) have wide applications in the military, health, and environmental fields [1,2,3]. Because node positions support information for many WSN applications, sensor localization has become a hot research issue. A new preventive routing method was proposed in [4] to improve energy consumption and increase throughput using location information. In [5], the location information of a mobile sensor is used for collaborative sleep scheduling in WSNs integrated with mobile cloud computing. The authors of [6] utilized the location information of a mobile robot to collect the sensed data from the partitioned/islanded WSNs. The authors of [6] collected sensed data from a WSN base station and improved their efficiency using the location information.

Many localization methods are presented in the literature [7,8,9,10,11,12,13,14,15,16,17,18,19,20,21,22,23,24,25,26,27,28,29,30,31,32]. There are two ways to locate a sensor in WSNs: (1) range-based and (2) range-free methods. The former utilizes time of arrival (TOA), time difference of arrival (TDOA), angle of arrival (AOA), received signal strength (RSS), or hybrid measurements to solve the nodes’ position. Since these algorithms use accurate measurement information, they usually obtain a high positioning accuracy. Compared with the range-based location method, the latter only uses connectivity information to locate a blindfolded node (BN), which reduces the hardware complexity at the cost of performance degradation.

Regarding range-based techniques, location methods were first proposed for Line-of-sight (LOS) situations. Several solutions [7,8,9,10], including closed-form algorithms based on least-squares (LS), weighted least-squares (WLS), maximum likelihood estimator (MLE), and the minimum mean square error estimator (MMSEE) criteria and iterative algorithms [11,12,13,14] based on Newton–Raphson and convex optimization criteria, are applied in range-based location techniques. The LS method [7] provides a suboptimal solution with the minimum amount of computation. Subsequently, the MLE method [8] based on two-step WLS solutions was proposed to attain the Cramer–Rao lower bound (CRLB). The MLE method based on a closed-form solution performs optimally with an acceptable computation complexity. Using the statistical characteristics of BN position as prior information, the MMSEE method [9] can further improve mobile location accuracy. In addition, more complicated cases, such as those considering the positioning errors of reference nodes (RNs) [14] and clock drift [15] among nodes, are studied in LOS localization algorithms. Due to the severe nonlinearity existing in some location techniques, such as RSS and hybrid methods, an MLE estimator may not lead to a closed-form solution. Various iterative algorithms, including Newton–Raphson and convex optimization methods [11,12,13,14], have been developed to obtain optimal performance. Besides LOS location methods, performance analysis in terms of CRLBs is also provided in the literature [16,17,18]. Non-line-of-sight (NLOS) presents a major problem in determining accurate location in WSNs, prompting the development of many positioning methods [19,20,21,22] and CRLBs [23,24,25] for NLOS propagation in the literature.

Compared with those on range-based techniques, relatively fewer studies [26,27,28,29,30,31,32] have been reported for range-free location techniques. Among range-free methods, Centroid-based localization (CL) is the most famous; it functions by determining the BN’s position as the centroid of RNs. To obtain a relative position estimate, a multidimensional scaling (MDS) algorithm based on the connectivity matrix was developed for range-free localization in a WSN [29]. Both References [26,27] analyzed the performance of range-free techniques. The former focused on the uniform distribution of RNs and derived a theoretical variance for the CL method. The latter assumed that the RNs’ position follows a Poisson point process, and the spatially averaged area of location region and localization error probability was derived based on this assumption.

**Figure 1:**
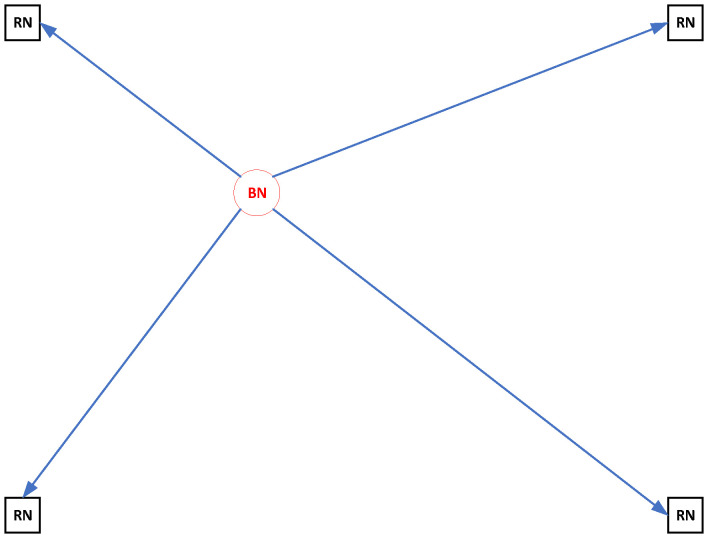
The traditional location technique using one-spot TOA measurements.

Although many studies are reported for both range-based and range-free location methods [7,8,9,10,11,12,13,14,15,16,17,18,19,20,21,22,23,24,25,26,27,28,29,30,31,32], they are all based on a one-spot measurement model, as shown in Figure 1. Since the existing location methods have been widely studied, it is difficult to improve their positioning accuracy further. Using a short-term motion model as prior information, this paper proposes two novel location methods with higher positioning accuracy than the existing location algorithms based on one-spot measurements. The two main contributions of this paper are listed as follows:

- This paper proposes two location methods, including closed-form and iterative solutions, to improve mobile location accuracy based on multi-spot measurements. Due to the increased number of measurement equations used in this paper, the performance of the proposed methods is better than that of traditional location methods using one-spot measurements. Simulations show that both proposed methods have a lower root mean square error (RMSE) than the traditional methods, and the proposed iterative method can attain the CRLB.
- A novel CRLB for the proposed multi-spot measurements model is also derived in this paper. This paper provides theoretical proof that the proposed CRLB is lower than the traditional CRLB. This implies that the multi-spot measurement model has higher positioning accuracy than the single-spot measurement model.

This paper is organized as follows. Section 2 briefly introduces the system model. A novel CRLB for the proposed multi-spot model is derived in Section 3. A closed-form solution based on a two-step WLS estimator is proposed in Section 4. To further improve the positioning accuracy, an iterative method is proposed in Section 5. In Section 6, the performance of the proposed algorithm is simulated in terms of the RMSE. The conclusions of this paper are given in Section 7.

## 2. System Model

The proposed methods are based on a TOA model introduced in this section. The position of a BN (x,y) is an unknown parameter. The known coordinate of the ith RN is set to be $\left(x_{i},y_{i}\right)$, and the number of RNs is N. Without a loss of generality, $(x_{1},y_{1})=\left(0,0\right)$. Although the TOA model is used in the proposed methods, a similar idea can also be applied to other location techniques. The measurement with the noise of $\left\{*\right\}$ is denoted as $\left\{\overset{⌢}{*}\right\}$. The true distance between the ith RN and BN can be modeled as:

(1) $$r_{i}^{2}={\left(x_{i}-x\right)}^{2}+{\left(y_{i}-y\right)}^{2}=k_{i}-2x_{i}x-2y_{i}y+k$$

where $k_{i}=x_{i}^{2}+y_{i}^{2}$ and $k=x^{2}+y^{2}$.

Traditional TOA location algorithms are based on one-spot TOA measurements collected from N-RNs, as shown in Figure 1.

With the TOA noise, the error vector derived from (1) is

(2) e=Y−GZ

where

$e={\left[\begin{array}{ccc} e_{1} & \cdots & e_{N} \end{array}\right]}^{T}$, $e_{i}={\overset{⌢}{r}}_{i}^{2}-r_{i}^{2}$,

$G=\left[\begin{array}{ccc} -2x_{1} & -2y_{1} & 1\\ \vdots & \ddots & \vdots \\ -2x_{N} & -2y_{N} & 1 \end{array}\right]$, $Y=\left[\begin{array}{c} {\overset{⌢}{r}}_{1}^{2}-k_{1}\\ \vdots \\ {\overset{⌢}{r}}_{N}^{2}-k_{N} \end{array}\right]$, $Z=\left[\begin{array}{c} Z_{1}\\ Z_{2}\\ Z_{3} \end{array}\right]=\left[\begin{array}{c} x\\ y\\ k \end{array}\right]$.

**Figure 2:**
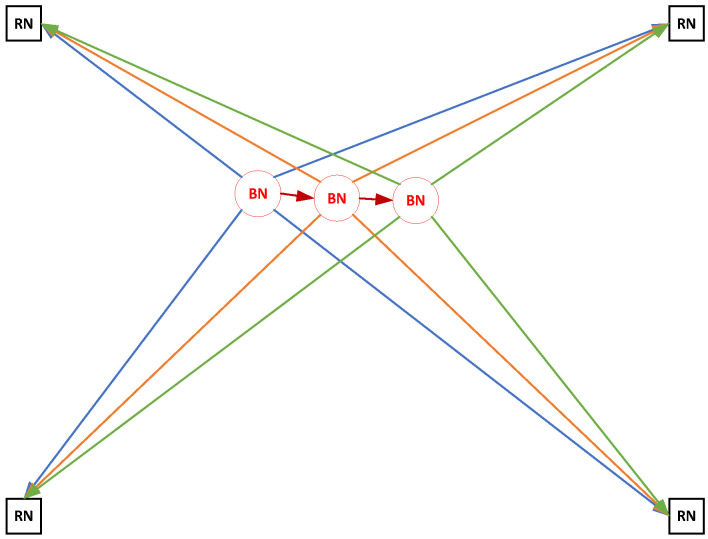
The proposed location technique using multi-spot TOA measurements.

Several estimators, such as MMSEE and MLE, are used in the traditional TOA-based location algorithms to solve (2). Compared with the existing location algorithms based on one-spot TOA measurements, multi-spot measurements are utilized in the proposed method to obtain a higher positioning accuracy, as presented in Figure 2. Based on the information theory, the more information there is on measurements, the greater the system gain, which is proved in the theoretical analysis detailed in Section 3.

In the case of multi-spot measurements, range data collected from multiple time moments and N-RNs can be modeled as follows:

(3) $$r_{ji}^{2}={\left(x_{i}-x_{j}^{’}\right)}^{2}+{\left(y_{i}-y_{j}^{’}\right)}^{2}=k_{i}-2x_{i}x_{j}^{’}-2y_{i}y_{j}^{’}+k_{j}^{’}$$

where $r_{ji}$ is the true distance between the ith RN and BN in the jth moment, i=1,⋯,N, and j=1,⋯,M. $\left(x_{j}^{’},y_{j}^{’}\right)$ is the position of a BN for the jth moment and $k_{j}^{’}={\left(x_{j}^{’}\right)}^{2}+{\left(y_{j}^{’}\right)}^{2}$.

The range measurement ${\overset{⌢}{r}}_{ji}$ can be modeled as:

(4) $${\overset{⌢}{r}}_{ji}=r_{ji}+n_{ji}$$

where $n_{ji}$ is the measurement noise, which is subject to a zero-mean Gaussian random process with a variance $\sigma_{ji}^{2}$.

Considering the case in which multiple range data $r_{ji}$ are collected within a short time period, such as several seconds, $\left(x_{j}^{’},y_{j}^{’}\right)$ can be modeled as a uniform linear motion. Thus, $\left(x_{j}^{’},y_{j}^{’}\right)$ can be calculated as:

(5) $$\begin{array}{l} x_{j}^{’}=x+v_{x}(j-1)T\\ y_{j}^{’}=y+v_{y}(j-1)T \end{array}$$

where (x,y) is the position of a BN at the first moment, and $v_{x}$ and $v_{y}$ are the BN speeds for the x and y axis, respectively. T is the measurement interval between two moments.

## 3. Cramer–Rao Lower Bound

It is well-known that the CRLB sets a lower limit for the variance or covariance matrix of any unbiased estimate of unknown parameters [33].

Although many CRLBs for various location techniques considering one-spot measurements have been presented in the literature [16,17,18,23,24,25], to the best of our knowledge, performance analysis for localization has not been carried out on a multi-spot measurement model in WSNs. This section provides performance analysis for localization using multi-spot measurements in terms of CRLB.

Let $\overset{⌢}{R}={\left[\begin{array}{ccc} {\overset{⌢}{R}}_{1}^{T} & \cdots & {\overset{⌢}{R}}_{M}^{T} \end{array}\right]}^{T}$ be a range measurement vector and θ be a parameter vector to be estimated, where ${\overset{⌢}{R}}_{j}={\left[\begin{array}{ccc} {\overset{⌢}{r}}_{j1} & \cdots & {\overset{⌢}{r}}_{jN} \end{array}\right]}^{T}$ and θ is ${\left[\begin{array}{cccc} x & y & v_{x} & v_{y} \end{array}\right]}^{T}$.

The CRLB is calculated based on the assumption that the probability density function (PDF) $f\left(\overset{⌢}{R};\theta \right)$ satisfies the “regularity” conditions, which are:

(6) $$E\left(\frac{\partial \ln f\left(\overset{⌢}{R};\theta \right)}{\partial \theta }\right)=0\;\text{for}\;\text{all}\;\theta$$

The CRLB matrix is defined as the inverse of the Fisher information matrix (FIM) $J_{\theta }$:

(7) $$E\left(\left(\overset{⌢}{\theta }-\theta \right){\left(\overset{⌢}{\theta }-\theta \right)}^{T}\right)\geq J_{\theta }^{-1}$$

where $\overset{⌢}{\theta }$ is an estimate of θ.

The FIM is determined by [33]:

(8) $$J_{\theta }=E\left[\frac{\partial \ln f\left(\overset{⌢}{R};\theta \right)}{\partial \theta }{\left(\frac{\partial \ln f\left(\overset{⌢}{R};\theta \right)}{\partial \theta }\right)}^{T}\right]$$

Using Bayes’ theorem,

(9) $$f\left(\overset{⌢}{R};\theta \right)=f\left(\overset{⌢}{R}|\theta \right)f\left(\theta \right)$$

Since θ is a deterministic unknown process, (9) becomes:

(10) $$f\left(\overset{⌢}{R};\theta \right)=f\left(\overset{⌢}{R}|\theta \right)$$

From (4) and (5), the PDF $f\left(\overset{⌢}{R}|\theta \right)$ can be written as:

(11) $$f\left(\overset{⌢}{R}|\theta \right)=\prod_{j=1}^{M}\prod_{i=1}^{N}f\left({\overset{⌢}{r}}_{ji}|\theta \right)$$

where

(12) $$\begin{array}{l} f\left({\overset{⌢}{r}}_{ji}|\theta \right)=\frac{1}{\sqrt{2\pi }\sigma_{ji}}\exp\left(-\frac{{\left({\overset{⌢}{r}}_{ji}-r_{ji}\right)}^{2}}{2\sigma_{ji}^{2}}\right)\\ r_{ji}=\sqrt{{\left(x_{i}-\left(x+v_{x}(j-1)T\right)\right)}^{2}+{\left(y_{i}-\left(y+v_{y}(j-1)T\right)\right)}^{2}} \end{array}$$

The log of $f\left(\overset{⌢}{R}|\theta \right)$ is:

(13) $$\begin{array}{l} \ln f\left(\overset{⌢}{R}|\theta \right)=\sum_{j=1}^{M}\sum_{i=1}^{N}\ln f\left({\overset{⌢}{r}}_{ji}|\theta \right)\\ =\sum_{j=1}^{M}\sum_{i=1}^{N}\ln\frac{1}{\sqrt{2\pi }\sigma_{ji}}-\sum_{j=1}^{M}\sum_{i=1}^{N}\frac{{\left({\overset{⌢}{r}}_{ji}-r_{ji}\right)}^{2}}{2\sigma_{ji}^{2}} \end{array}$$

Substituting (13) into $\partial \ln f\left(\overset{⌢}{R}|\theta \right)/\partial \theta_{k}$ gives:

(14) $$\frac{\partial \ln f\left(\overset{⌢}{R}|\theta \right)}{\partial \theta_{k}}=\sum_{j=1}^{M}\sum_{i=1}^{N}\frac{\left({\overset{⌢}{r}}_{ji}-r_{ji}\right)}{\sigma_{ji}^{2}}\frac{\partial r_{ji}}{\partial \theta_{k}}=\sum_{j=1}^{M}\sum_{i=1}^{N}\frac{n_{ji}}{\sigma_{ji}^{2}}\frac{\partial r_{ji}}{\partial \theta_{k}}$$

where

(15) $$\begin{array}{l} \frac{\partial r_{ji}}{\partial x}=\frac{\left(x+v_{x}(j-1)T\right)-x_{i}}{r_{ji}},\frac{\partial r_{ji}}{\partial y}=\frac{\left(y+v_{y}(j-1)T\right)-y_{i}}{r_{ji}},\\ \frac{\partial r_{ji}}{\partial v_{x}}=\frac{\left(\left(x+v_{x}(j-1)T\right)-x_{i}\right)(j-1)T}{r_{ji}},\\ \frac{\partial r_{ji}}{\partial v_{y}}=\frac{\left(\left(y+v_{y}(j-1)T\right)-y_{i}\right)(j-1)T}{r_{ji}} \end{array}$$

Since $E\left[n_{ji}\right]=0$, the expectation of $\partial \ln f\left(\overset{⌢}{R}|\theta \right)/\partial \theta_{k}$ is:

(16) $$E\left[\partial \ln f\left(\overset{⌢}{R}|\theta \right)/\partial \theta_{k}\right]=0$$

Compared with (6) and (16), it is observed that $f\left(\overset{⌢}{R};\theta \right)$ satisfies the “regularity” conditions, indicating the CRLB exists in the case of multi-spot measurements.

Substituting (14) and (15) into (8) gives:

(17) $$J_{\theta }=HQ^{-1}H^{T}$$

where

(18) $$\begin{array}{l} H=\left[\begin{array}{ccc} H_{1} & \cdots & H_{M} \end{array}\right],Q=diag\left\{\left[\begin{array}{ccc} Q_{1} & \cdots & Q_{M} \end{array}\right]\right\}\\ H_{j}=\left[\begin{array}{ccc} \frac{\partial r_{j1}}{\partial x} & \cdots & \frac{\partial r_{jN}}{\partial x}\\ \frac{\partial r_{j1}}{\partial y} & \cdots & \frac{\partial r_{jN}}{\partial y}\\ \frac{\partial r_{j1}}{\partial v_{x}} & \cdots & \frac{\partial r_{jN}}{\partial v_{x}}\\ \frac{\partial r_{j1}}{\partial v_{y}} & \cdots & \frac{\partial r_{jN}}{\partial v_{y}} \end{array}\right],Q_{j}=diag\left\{\left[\begin{array}{ccc} \sigma_{j1}^{2} & \cdots & \sigma_{jN}^{2} \end{array}\right]\right\}. \end{array}$$

Considering a general case in which $\sigma_{ji}=\sigma$, (17) can be rewritten as:

(19) $$J_{\theta }=\frac{1}{\sigma^{2}}HH^{T}=\frac{1}{\sigma^{2}}\sum_{j=1}^{M}H_{j}H_{j}^{T}$$

The relationship between the prosed CRLB for the case of multi-spot measurements and the traditional CRLB for the case of single-spot measurements is provided in the following proposition.

**Proposition** **1.** *In the TOA localization technique, the traditional CRLB for the case of single-spot measurements is higher than the prosed CRLB for the case of multi-spot measurements.*

(20) $$tr\left\{{\left(J_{p}^{-1}\right)}_{2\times 2}\right\}=tr\left\{{\left({\left(\frac{\sum_{j=1}^{M}H_{j}H_{j}^{T}}{\sigma^{2}}\right)}^{-1}\right)}_{{}_{2\times 2}}\right\}\leq tr\left\{J_{t}^{-1}\right\}=tr\left\{{\left(\frac{MM^{T}}{\sigma^{2}}\right)}^{-1}\right\}$$

*where*$J_{p}$*and*$J_{t}$*are the FIMs for the proposed and traditional CRLBs*, respectively*. From* [34]*, it can be found that*

(21) $$M=\left[\begin{array}{ccc} \frac{x}{r_{11}} & \cdots & \frac{x}{r_{1N}}\\ \frac{y}{r_{11}} & \cdots & \frac{y}{r_{1N}} \end{array}\right]$$

**Proof** **of** **Proposition** **1.** For a positive semi-definite matrix B, it is proved in [24] that:

(22) $$tr\left\{{\left(A+B\right)}^{-1}\right\}\leq tr\left\{A^{-1}\right\}$$

Obviously, $\sum_{j=2}^{M}H_{j}H_{j}^{T}$ is a positive semi-definite matrix. Thus, the following inequation holds:

(23) $$tr\left\{{\left({\left(\frac{\sum_{j=1}^{M}H_{j}H_{j}^{T}}{\sigma^{2}}\right)}^{-1}\right)}_{{}_{2\times 2}}\right\}\leq tr\left\{{\left({\left(\frac{H_{1}H_{1}^{T}}{\sigma^{2}}\right)}^{-1}\right)}_{{}_{2\times 2}}\right\}$$

Let j=1, so $H_{1}$ in (18) becomes:

(24) $$H_{1}=\left[\begin{array}{ccc} \frac{x}{r_{11}} & \cdots & \frac{x}{r_{1N}}\\ \frac{y}{r_{11}} & \cdots & \frac{y}{r_{1N}}\\ 0 & \cdots & 0\\ 0 & \cdots & 0 \end{array}\right]=\left[\begin{array}{c} M\\ 0_{2\times N} \end{array}\right]$$

where $0_{2\times N}$ is the zero matrix with two rows and N columns.

Substituting (24) into $H_{1}H_{1}^{T}$gives:

(25) $$H_{1}H_{1}^{T}=\left[\begin{array}{cc} MM^{T} & 0_{2\times 2}\\ 0_{2\times 2} & 0_{2\times 2} \end{array}\right]$$

From (25),

(26) $$tr\left\{{\left({\left(\frac{H_{1}H_{1}^{T}}{\sigma^{2}}\right)}^{-1}\right)}_{{}_{2\times 2}}\right\}=tr\left\{{\left(\frac{MM^{T}}{\sigma^{2}}\right)}^{-1}\right\}$$

Substituting (26) into (23), Proposition 1 holds. □

The CRLB is a popular tool for performance analysis since it determines the physical impossibility of the variance of an unbiased estimator being less than the bound. Unfortunately, the CRLB cannot be used for arbitrary noise distribution. For a CRLB, the PDF $f\left(\overset{⌢}{R};\theta \right)$ must satisfy the “regularity” conditions. This limitation determines that a CRLB usually exists under Gaussian noise conditions. For other noise distributions, such as uniformly distributed noise, a CRLB may not exist.

## 4. The Proposed Method with a Closed-Form Solution

The proposed method uses multi-spot measurements (3) and a BN motion model (5) to improve positioning accuracy. It can be seen from (3) and (5) that the unknown parameters to be estimated are $\left(x,y,v_{x},v_{y}\right)$, and the number of measurement equation (3) is N∗M. For a 2D localization problem, *N* must be equal to or greater than 3. Thus, the measurement equation (3) can obtain a solution for M≥2. This indicates that the proposed method can be applied in most situations.

Substituting (5) into $k_{j}^{’}$ gives:

(27) $$\begin{array}{l} k_{j}^{’}={\left(x+v_{x}(j-1)T\right)}^{2}+{\left(y+v_{y}(j-1)T\right)}^{2}\\ \;=x^{2}+2xv_{x}(j-1)T+{\left(v_{x}(j-1)T\right)}^{2}\\ \;+y^{2}+2yv_{y}(j-1)T+{\left(v_{y}(j-1)T\right)}^{2}\\ \;=u_{1}+{\left((j-1)T\right)}^{2}u_{2}+2(j-1)Tu_{3} \end{array}$$

where $u_{1}=x^{2}+y^{2}$, $u_{2}=v_{x}^{2}+v_{y}^{2}$, and $u_{3}=xv_{x}+yv_{y}$.

Substituting (5) into (3) gives:

(28) $$r_{ji}^{2}=k_{i}-2x_{i}\left(x+v_{x}(j-1)T\right)-2y_{i}\left(y+v_{y}(j-1)T\right)+k_{j}^{’}$$

Substituting (27) into (28) gives:

(29) $$\begin{array}{c} r_{ji}^{2}-k_{i}=-2x_{i}x-2y_{i}y-2x_{i}(j-1)Tv_{x}-2y_{i}(j-1)T\\ u_{1}+{\left((j-1)T\right)}^{2}u_{2}+2(j-1)Tu_{3} \end{array}$$

It can be seen that Equation (29) becomes a linear equation when three new unknown variables $\left(u_{1},u_{2},u_{3}\right)$ are introduced. Although the linearization method may need more range measurements to obtain a unique solution, it permits the proposed method to be applied for real-time implementation with the closed-form solution.

The matrix form of (29) can be rewritten as:

(30) e=Y−GZ

where

$e={\left[\begin{array}{ccc} e_{1}^{T} & \cdots & e_{M}^{T} \end{array}\right]}^{T}$, $e_{j}={\left[\begin{array}{ccc} {\hat{r}}_{j1}^{2}-r_{j1}^{2} & \cdots & {\hat{r}}_{jN}^{2}-r_{jN}^{2} \end{array}\right]}^{T}$,

$\begin{array}{l} Z={\left[\begin{array}{ccccccc} Z_{1} & Z_{2} & Z_{3} & Z_{4} & Z_{5} & Z_{6} & Z_{7} \end{array}\right]}^{T}\\ \;={\left[\begin{array}{ccccccc} x & y & v_{x} & v_{y} & u_{1} & u_{2} & u_{3} \end{array}\right]}^{T} \end{array}$, $Y={\left[\begin{array}{ccc} Y_{1}^{T} & \cdots & Y_{M}^{T} \end{array}\right]}^{T}$,

(31) $$\begin{array}{l} Y_{j}={\left[\begin{array}{ccc} {\overset{⌢}{r}}_{j1}^{2}-k_{1} & \cdots & {\overset{⌢}{r}}_{jN}^{2}-k_{N} \end{array}\right]}^{T},G={\left[\begin{array}{ccc} G_{1}^{T} & \cdots & G_{M}^{T} \end{array}\right]}^{T},\\ G_{j}=\left[\begin{array}{ccccccc} -2x_{1} & -2y_{1} & -2x_{1}\left(j-1\right)T & -2y_{1}\left(j-1\right)T & 1 & {\left((j-1)T\right)}^{2} & 2\left(j-1\right)T\\ \vdots & \vdots & \vdots & \vdots & \vdots & \vdots & \vdots \\ -2x_{N} & -2y_{N} & -2x_{N}\left(j-1\right)T & -2y_{N}\left(j-1\right)T & 1 & {\left((j-1)T\right)}^{2} & 2\left(j-1\right)T \end{array}\right] \end{array},\;j=1,\cdots ,M$$

Both LS and WLS can be used to solve (30). For an LS estimator, the unknown vector Z is calculated as:

$$Z={\left(G^{T}G\right)}^{-1}G^{T}Y$$

Since the error vector e contains different error variances, higher positioning accuracy of (30) can be obtained using the WLS estimator.

The WLS estimator of Z can be obtained from (30):

(32) $$\begin{array}{l} Z=\arg\min\left\{{\left(Y-GZ\right)}^{T}\Psi^{-1}\left(Y-GZ\right)\right\}\\ \;={\left(G^{T}\Psi^{-1}G\right)}^{-1}G^{T}\Psi^{-1}Y \end{array}$$

where ψ is the covariance matrix of e:

(33) $$\psi =\mathrm{cov}\left(e\right)=E\left(ee^{T}\right)$$

Ignoring the square error term and being derived from (30), the element $e_{ji}$ of e can be expressed as:

(34) $$\begin{array}{l} e_{ji}={\hat{r}}_{ji}^{2}-r_{ji}^{2}={\left(r_{ji}+n_{ji}\right)}^{2}-r_{ji}^{2}\\ \;=r_{ji}^{2}+2r_{ji}n_{ji}+n_{ji}^{2}-r_{ji}^{2}\\ \;\approx 2r_{ji}n_{ji} \end{array}$$

From (34), the expectations of $e_{ji}^{2}$ can be obtained as follows:

(35) $$E\left(e_{ji}^{2}\right)=E\left[{\left(2r_{ji}n_{ji}\right)}^{2}\right]={\left(2r_{ji}\right)}^{2}E\left[n_{ji}^{2}\right]={\left(2r_{ji}\right)}^{2}\sigma_{ji}^{2}$$

The expectations of $e_{ji}e_{mn}$(m≠j or n≠i) are:

(36) $$\begin{array}{c} E\left(e_{ji}e_{mn}\right)=E\left[2r_{ji}n_{ji}2r_{mn}n_{mn}\right]=4r_{ji}r_{mn}E\left[n_{ji}n_{mn}\right]\\ =4r_{ji}r_{mn}E\left[n_{ji}\right]E\left[n_{mn}\right]=0 \end{array}$$

Substituting (35) and (36) into (33) gives:

(37) $$\psi =\mathrm{cov}\left(e\right)=E\left(ee^{T}\right)=BQB$$

where

(38) $$\begin{array}{l} B=diag\left\{\left[\begin{array}{ccc} B_{1} & \cdots & B_{M} \end{array}\right]\right\},B_{j}=diag\left\{\left[\begin{array}{ccc} 2r_{j1} & \cdots & 2r_{jN} \end{array}\right]\right\}\\ Q=diag\left\{\left[\begin{array}{ccc} Q_{1} & \cdots & Q_{M} \end{array}\right]\right\},Q_{j}=diag\left\{\left[\begin{array}{ccc} \sigma_{j1}^{2} & \cdots & \sigma_{jN}^{2} \end{array}\right]\right\} \end{array}$$

The above equation shows that the covariance matrix ψ depends on the unknown $r_{ji}$. A further approximation is necessary to make the problem solvable. A common method is to replace the true range $r_{ji}$ with the range measurement ${\hat{r}}_{ji}$ in ψ.

The covariance matrix of Z can be calculated using the perturbation approach. Δ is denoted as the error perturbation. Obviously, neither G or ψ contain noise, whereas the range measurement ${\hat{r}}_{j1}^{2}$ exists in the Y. Thus,

(39) $$\begin{array}{c} \Delta Z={\left(G^{T}\Psi^{-1}G\right)}^{-1}G^{T}\Psi^{-1}\Delta Y\\ ={\left(G^{T}\Psi^{-1}G\right)}^{-1}G^{T}\Psi^{-1}e \end{array}$$

Substituting (39) into $\mathrm{cov}\left(Z\right)$, the covariance matrix of Z can be obtained:

(40) $$\begin{array}{l} \mathrm{cov}\left(Z\right)=E\left[\Delta Z\Delta Z^{T}\right]\\ \;\;\;={\left(G^{T}\Psi^{-1}G\right)}^{-1}G^{T}\Psi^{-1}E\left[ee^{T}\right]\Psi^{-1}G{\left(G^{T}\Psi^{-1}G\right)}^{-1}\\ \;\;\;={\left(G^{T}\Psi^{-1}G\right)}^{-1}G^{T}\Psi^{-1}\Psi \Psi^{-1}G{\left(G^{T}\Psi^{-1}G\right)}^{-1}\\ \;\;\;={\left(G^{T}\Psi^{-1}G\right)}^{-1} \end{array}$$

Based on the assumption that the unknown parameters to be estimated, $Z_{i}$, are independent, the WLS estimator (32) is an MLE and can attain the optimal performance. Unfortunately, those parameters are correlated since the linearization method is used for (29) to allow (30) to have a closed-form solution. The estimation accuracy can be further improved using the relationship among $Z_{i}$. The results can be revised as follows using the relations $u_{1}=x^{2}+y^{2}$ and $u_{2}=v_{x}^{2}+v_{y}^{2}$:

(41) e’=Y’−G’Z’

where

(42) $$Y’=\left[\begin{array}{c} Z_{1}^{2}\\ Z_{2}^{2}\\ Z_{3}^{2}\\ Z_{4}^{2}\\ Z_{5}\\ Z_{6} \end{array}\right],\;G’=\left[\begin{array}{cccc} 1 & 0 & 0 & 0\\ 0 & 1 & 0 & 0\\ 0 & 0 & 1 & 0\\ 0 & 0 & 0 & 1\\ 1 & 1 & 0 & 0\\ 0 & 0 & 1 & 1 \end{array}\right],\;Z’=\left[\begin{array}{c} x^{2}\\ y^{2}\\ v_{x}^{2}\\ v_{y}^{2} \end{array}\right].\;$$

assuming that the estimation errors of x, y, $v_{x}$, $v_{y}$, $u_{1}$ and $u_{2}$ are $\eta_{1}$, $\eta_{2}$, $\eta_{3}$, $\eta_{4}$, $\eta_{5}$ and $\eta_{6}$, respectively.

Then, the elements of Z become:

(43) $$Z_{1}=x+\eta_{1},\;Z_{2}=y+\eta_{2},\;Z_{3}=v_{x}+\eta_{3}\;Z_{4}=v_{y}+\eta_{4},Z_{5}=u_{1}+\eta_{5},\;Z_{6}=u_{2}+\eta_{6}$$

Substituting (43) into (41) and ignoring the square error term, the entries of e’ can be expressed as:

(44) $$e{’}_{1}=2x\mu_{1},\;e{’}_{2}=2y\mu_{2},\;e{’}_{3}=2v_{x}\eta_{3}\;e{’}_{4}=2v_{y}\eta_{4},\;e{’}_{5}=\eta_{5},\;e{’}_{3}=\eta_{6}$$

Subsequently, the covariance matrix of e’ is:

(45) $$\Psi ’=E\left(e’e{’}^{T}\right)=B’{\left\{\mathrm{cov}\left(Z\right)\right\}}_{\left(1:6\right)\times \left(1:6\right)}B’$$

where $B’=diag\left\{\left[2x,2y,2v_{x},2v_{y},1,1\right]\right\}$. In fact, B’ is unknown as B’ contains the true BN position x and y. B’ can be approximated as $B’=diag\left\{\left[2Z_{1},2Z_{2},2Z_{3},2Z_{4},1,1\right]\right\}$. $\mathrm{cov}\left(Z\right)$ is the covariance matrix of Z and can be calculated using (40).

The second step of the WLS solution is:

(46) $$Z’={\left(G{’}^{T}\Psi {’}^{-1}G’\right)}^{-1}G{’}^{T}\Psi {’}^{-1}Y’$$

Similarly, the covariance matrix of Z’ can be obtained using the perturbation approach:

(47) $$\mathrm{cov}\left(Z’\right)={\left(G{’}^{T}\Psi {’}^{-1}G’\right)}^{-1}$$

The position estimation of Z’’ is obtained as follows:

(48) $$Z’’=sign\left(Z\right)\sqrt{Z’}$$

In summary, the steps of the proposed method are as follows:

(1) Estimate ψ through substituting (38) into (37).
(2) The first weight solution of BN can be obtained by substituting (37) into (32).
(3) The final solution of BN can be obtained from (48).

From the definition of Z’ in (42) and ignoring the square error term, Z’ can be rewritten as:

(49) $$Z{’}_{1}-x^{2}=2xe_{x},\;Z{’}_{2}-y^{2}=2ye_{y}\;Z{’}_{3}-v_{x}^{2}=2v_{x}e_{vx},\;Z{’}_{4}-v_{y}^{2}=2v_{y}e_{vy}$$

where $e_{x}$, $e_{y}$, $e_{vx}$ and $e_{vy}$ are the estimation errors of x, y, $v_{x}$ and $v_{y}$ in (44), respectively. The covariance matrix of Z’’ can be obtained from (48):

(50) $$\mathrm{cov}\left(Z’’\right)=B’{’}^{-1}\mathrm{cov}(Z’)B’{’}^{-1}$$

where $B’’=2diag\left\{\left[\begin{array}{cccc} x & y & v_{x} & v_{y} \end{array}\right]\right\}$.

From (40), (45), (47), and (48), the covariance matrix of Z’’ can finally be obtained:

(51) $$\begin{array}{l} \mathrm{cov}\left(Z’’\right)={\left(B’’\mathrm{cov}{\left(Z’\right)}^{-1}B’’\right)}^{-1}\\ \;\;\;\;={\left(B’’G{’}^{T}\Psi {’}^{-1}G’B’’\right)}^{-1}\\ \;\;\;\;={\left(B’’G{’}^{T}B{’}^{-1}{\left\{\mathrm{cov}{\left(Z\right)}_{\left(1:6\right)\times \left(1:6\right)}\right\}}^{-1}B{’}^{-1}G’B’’\right)}^{-1} \end{array}$$

## 5. The Proposed Method with Iterative Solution

The proposed closed-form solution in Section 4 attempts to provide an optimal performance using a two-step WLS estimator. Due to the severe nonlinearity, the relationship $u_{3}=xv_{x}+yv_{y}$ is not used in the second WLS solution (41), which may lead to suboptimal performance. In this section, an iterative Newton–Raphson method based on an MLE is developed to obtain a higher positioning accuracy.

The MLE is found by maximizing the PDF (11) or, equivalently, by maximizing the likelihood function.

(52) $$J\left(\theta \right)=\ln f\left(\overset{⌢}{R}|\theta \right)=\sum_{j=1}^{M}\sum_{i=1}^{N}\ln\frac{1}{\sqrt{2\pi }\sigma_{ji}}-\sum_{j=1}^{M}\sum_{i=1}^{N}\frac{{\left({\overset{⌢}{r}}_{ji}-r_{ji}\right)}^{2}}{2\sigma_{ji}^{2}}$$

The iterative method attempts to maximize the likelihood function (52) by finding a zero of the derivative function. Using $\partial J\left(\theta \right)/\partial \theta =0$,

(53) $$g_{1}\left(\theta \right)=\sum_{j=1}^{M}\sum_{i=1}^{N}\frac{\gamma \left(\theta \right)}{\sigma_{ji}^{2}r_{ji}}=0,\;g_{2}\left(\theta \right)=\sum_{j=1}^{M}\sum_{i=1}^{N}\frac{\kappa \left(\theta \right)}{\sigma_{ji}^{2}r_{ji}}=0,\;g_{3}\left(\theta \right)=\sum_{j=1}^{M}\sum_{i=1}^{N}\frac{(j-1)T\gamma \left(\theta \right)}{\sigma_{ji}^{2}r_{ji}}=0,\;g_{4}\left(\theta \right)=\sum_{j=1}^{M}\sum_{i=1}^{N}\frac{(j-1)T\kappa \left(\theta \right)}{\sigma_{ji}^{2}r_{ji}}=0.\;$$

where

(54) $$\gamma \left(\theta \right)=\left({\overset{⌢}{r}}_{ji}-r_{ji}\right)\left(\left(x+v_{x}(j-1)T\right)-x_{i}\right)\;\kappa \left(\theta \right)=\left({\overset{⌢}{r}}_{ji}-r_{ji}\right)\left(\left(y+v_{y}(j-1)T\right)-y_{i}\right)$$

assuming that there is an initial guess for the solution to (53). Using the first-order approximation of the Taylor expansion, (53) becomes:

(55) $$\begin{array}{r} g_{i}\left(\theta \right)\approx g_{i}\left(\theta_{0}\right)+\frac{\partial g_{i}\left(\theta \right)}{\partial x}\left|{}_{\theta =\theta_{0}}\left(x-x_{0}\right)\right.+\frac{\partial g_{i}\left(\theta \right)}{\partial y}\left|{}_{\theta =\theta_{0}}\left(y-y_{0}\right)\right.\\ +\frac{\partial g_{i}\left(\theta \right)}{\partial v_{x}}\left|{}_{\theta =\theta_{0}}\left(v_{x}-v_{x0}\right)\right.+\frac{\partial g_{i}\left(\theta \right)}{\partial v_{y}}\left|{}_{\theta =\theta_{0}}\left(v_{y}-v_{y0}\right)\right.\approx 0\\ i=1,2,3,4 \end{array}$$

where

(56) $$\frac{\partial g_{1}\left(\theta \right)}{\partial \theta_{i}}=\sum_{j=1}^{M}\sum_{i=1}^{N}\frac{1}{\sigma_{ji}^{2}}\frac{\partial \gamma \left(\theta \right)/\partial \theta_{i}r_{ji}-\gamma \left(\theta \right)\partial r_{ji}/\partial \theta_{i}}{r_{ji}^{2}}\;\frac{\partial g_{2}\left(\theta \right)}{\partial \theta_{i}}=\sum_{j=1}^{M}\sum_{i=1}^{N}\frac{1}{\sigma_{ji}^{2}}\frac{\partial \kappa \left(\theta \right)/\partial \theta_{i}r_{ji}-\kappa \left(\theta \right)\partial r_{ji}/\partial \theta_{i}}{r_{ji}^{2}}\;\frac{\partial g_{3}\left(\theta \right)}{\partial \theta_{i}}=\sum_{j=1}^{M}\sum_{i=1}^{N}\frac{(j-1)T}{\sigma_{ji}^{2}}\frac{\partial \gamma \left(\theta \right)/\partial \theta_{i}r_{ji}-\gamma \left(\theta \right)\partial r_{ji}/\partial \theta_{i}}{r_{ji}^{2}}\;\frac{\partial g_{4}\left(\theta \right)}{\partial \theta_{i}}=\sum_{j=1}^{M}\sum_{i=1}^{N}\frac{(j-1)T}{\sigma_{ji}^{2}}\frac{\partial \kappa \left(\theta \right)/\partial \theta_{i}r_{ji}-\kappa \left(\theta \right)\partial r_{ji}/\partial \theta_{i}}{r_{ji}^{2}}$$

with

(57) $$\frac{\partial \gamma \left(\theta \right)}{\partial x}={\overset{⌢}{r}}_{ji}-\left(\frac{\partial r_{ji}}{\partial x}x+r_{ji}\right)-v_{x}(j-1)T\frac{\partial r_{ji}}{\partial x}\;\frac{\partial \gamma \left(\theta \right)}{\partial y}=-\frac{\partial r_{ji}}{\partial y}x-v_{x}(j-1)T\frac{\partial r_{ji}}{\partial y}\;\frac{\partial \gamma \left(\theta \right)}{\partial v_{x}}={\overset{⌢}{r}}_{ji}(j-1)T-x\frac{\partial r_{ji}}{\partial v_{x}}-(j-1)T\left(\frac{\partial r_{ji}}{\partial v_{x}}v_{x}+r_{ji}\right)\;\frac{\partial \gamma \left(\theta \right)}{\partial v_{y}}=-\frac{\partial r_{ji}}{\partial v_{y}}x-v_{x}(j-1)T\frac{\partial r_{ji}}{\partial v_{y}}\;\frac{\partial \kappa \left(\theta \right)}{\partial x}=-\frac{\partial r_{ji}}{\partial x}y-v_{y}(j-1)T\frac{\partial r_{ji}}{\partial x}\;\frac{\partial \kappa \left(\theta \right)}{\partial y}={\overset{⌢}{r}}_{ji}-\left(\frac{\partial r_{ji}}{\partial y}y+r_{ji}\right)-v_{y}(j-1)T\frac{\partial r_{ji}}{\partial y}\;\frac{\partial \kappa \left(\theta \right)}{\partial v_{x}}=-\frac{\partial r_{ji}}{\partial v_{x}}y-v_{y}(j-1)T\frac{\partial r_{ji}}{\partial v_{x}}\;\frac{\partial \kappa \left(\theta \right)}{\partial v_{y}}={\overset{⌢}{r}}_{ji}(j-1)T-y\frac{\partial r_{ji}}{\partial v_{y}}-(j-1)T\left(\frac{\partial r_{ji}}{\partial v_{y}}v_{y}+r_{ji}\right)$$

$\theta_{0}={\left[\begin{array}{cccc} x_{0} & y_{0} & v_{x0} & v_{y0} \end{array}\right]}^{T}$ is the initial guess, which can be calculated from the closed-form solution (48), and $\partial r_{ji}/\partial \theta_{i}$ can be computed using (15).

Expressing (55) in matrix form gives:

(58) $$F\left(\theta_{k+1}-\theta_{k}\right)=-P$$

where $\theta_{k}$ is the kth iterative estimate of θ.

(59) $$F=\left[\begin{array}{cccc} \frac{\partial g_{1}\left(\theta \right)}{\partial x} & \frac{\partial g_{1}\left(\theta \right)}{\partial y} & \frac{\partial g_{1}\left(\theta \right)}{\partial v_{x}} & \frac{\partial g_{1}\left(\theta \right)}{\partial v_{y}}\\ \frac{\partial g_{2}\left(\theta \right)}{\partial x} & \frac{\partial g_{2}\left(\theta \right)}{\partial y} & \frac{\partial g_{2}\left(\theta \right)}{\partial v_{x}} & \frac{\partial g_{2}\left(\theta \right)}{\partial v_{y}}\\ \frac{\partial g_{3}\left(\theta \right)}{\partial x} & \frac{\partial g_{3}\left(\theta \right)}{\partial y} & \frac{\partial g_{3}\left(\theta \right)}{\partial v_{x}} & \frac{\partial g_{3}\left(\theta \right)}{\partial v_{y}}\\ \frac{\partial g_{4}\left(\theta \right)}{\partial x} & \frac{\partial g_{4}\left(\theta \right)}{\partial y} & \frac{\partial g_{4}\left(\theta \right)}{\partial v_{x}} & \frac{\partial g_{4}\left(\theta \right)}{\partial v_{x}} \end{array}\right]\left|{}_{\theta =\theta_{k}}\right.\;P={\left[\begin{array}{ccc} g_{1}\left(\theta_{k}\right) & \cdots & g_{4}\left(\theta_{k}\right) \end{array}\right]}^{T}$$

Since P is the derivate of the log-likelihood function, we find the MLE as follows:

(60) $$\theta_{k+1}=\theta_{k}-{\left(F^{T}F\right)}^{-1}F^{T}P$$

Note that, at the convergence of $\theta_{k+1}=\theta_{k}$, and from (55) $g_{1}\left(\theta_{k}\right)=g_{2}\left(\theta_{k}\right)=g_{3}\left(\theta_{k}\right)=g_{4}\left(\theta_{k}\right)=0$, as desired. To avoid the divergency of the proposed iterative method, the closed-form solution (48) obtained from the two-step WLS is used as the initial value for the iterative method.

## 6. Simulation Results

**Figure 3:**
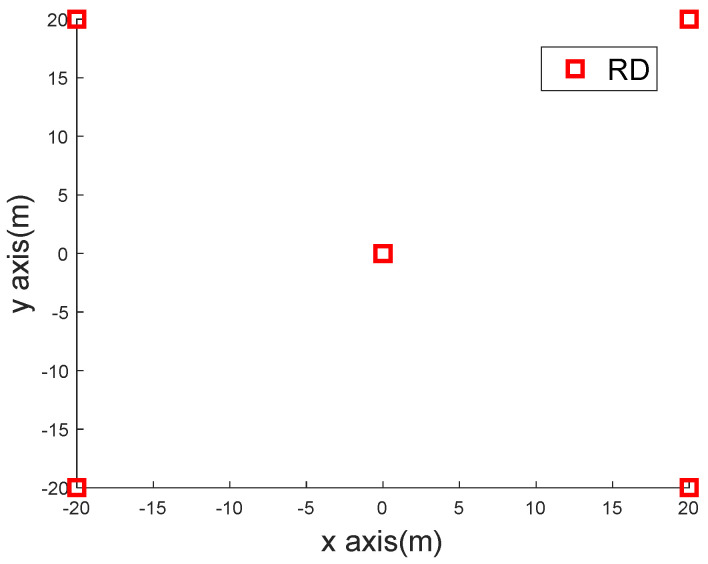
The topology diagram of the RN distribution.

The simulations considered a square 40 m × 40 m region. Both the position and speed of BN were assumed to follow the uniformly distributed random processes with −20≤x,y≤20 m and $-5\leq v_{x},v_{y}\leq 5$ m/s. (0, 0) m, (−20, 20) m, (20, 20) m, (20, −20) m, and (−20, 20) m were the coordinates of the RNs. A topology diagram of the RN distribution is shown in Figure 3. The measurement interval T was set to be 0.5 s in the simulation.

The RMSEs are defined as $\sqrt{E\left[{\left(x-\overset{⌢}{x}\right)}^{2}+{\left(y-\overset{⌢}{y}\right)}^{2}\right]}$ in the units of m and were obtained from the average of 5000 independent runs.

**Figure 4:**
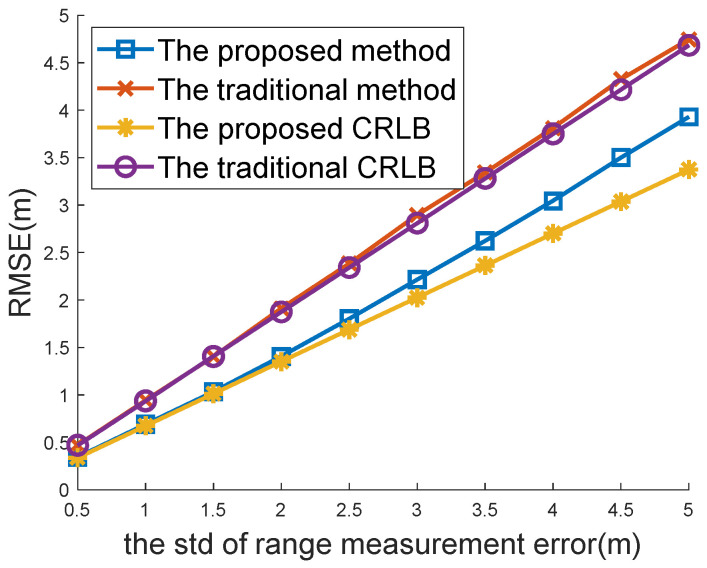
Comparison between the traditional method and the proposed method under different TOA noises.

**Figure 5:**
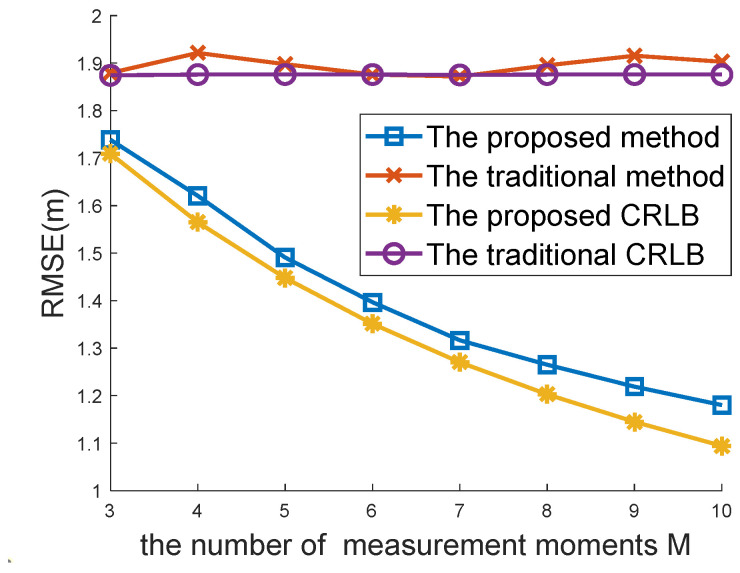
Comparison between the traditional method and the proposed method under different Ms.

Figure 4 and Figure 5 compare the positioning accuracy between the traditional location algorithm for the case of one-spot TOA measurements and the proposed method for the case of multi-spot measurements. Both the traditional WLS estimator [8,34] with a closed-form solution and the CRLB [34] are compared in Figure 4 and Figure 5 with the proposed closed-form solution (48) and CRLB (19).

The RMSEs versus the standard deviations (STDs) σ of range measurement errors are plotted in Figure 4. The number of multi-spot measurements was M=6 in this simulation. It was observed that both the proposed location algorithm and the proposed CRLB performed better than the traditional location and CRLB. With the increasing range of measurement error, the proposed method had more obvious advantages.

Performance comparisons with different Ms are recorded in Figure 5. The measurement error range σ was set to 2 m in this simulation. The proposed method using multi-spot measurements considerably outperformed the traditional method in the case of single-spot measurements. As M increased, the performance of the proposed method and CRLB improved. In contrast, the traditional method and CRLB remained unchanged since multi-spot measurements were used to improve positioning accuracy.

The RMSE curve of the traditional method was more volatile than that of the proposed method, as presented in Figure 5. This indicates that a greater number of measurements collected from multi-spot measurements can help to maintain system stability.

Figure 4 and Figure 5 show that the proposed CRLB was smaller than the traditional CRLB. This finding is in agreement with Proposition 1, which proves its effectiveness.

The following simulations were performed to observe how the proposed methods would perform with different estimators. Those estimators included the LS solution (32), the two-step WLS solution (48), and the iterative solution (60). The former two have a closed-form solution, whereas the final one needs an iterative search process.

**Figure 6:**
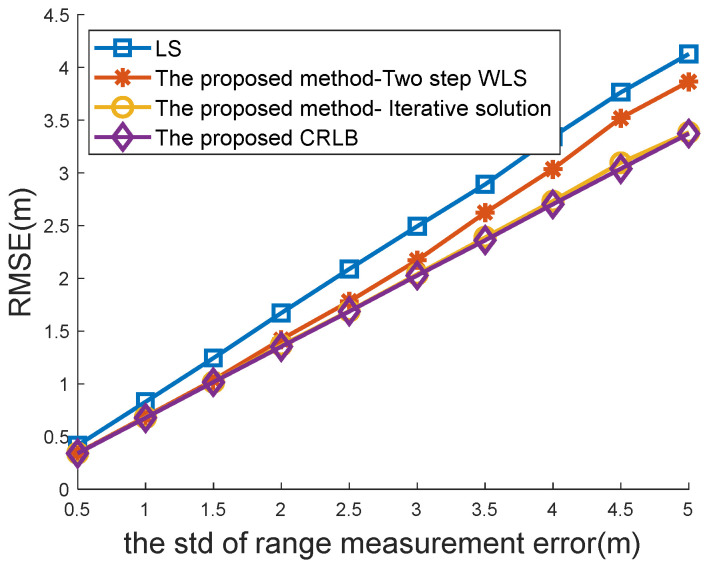
Comparison of the proposed methods under different TOA noises.

Figure 6 shows the RMSEs’ versus the STDs σ of the range measurement errors when M=6. It can be seen from the figure that both the proposed methods with WLS and iterative estimators performed better than the LS estimator. Among those estimators, the iterative method of Newton–Raphson had the best performance and could attain the CRLB. Although the proposed WLS method attempted to achieve optimal performance with the closed-form solution, there was a slight gap between the proposed WLS method and the CRLB under the condition of large measurement noise. This is because only two relations, $u_{1}=x^{2}+y^{2}$ and $u_{2}=v_{x}^{2}+v_{y}^{2}$, are used in the proposed WLS method (41) to improve positioning accuracy. The relation $u_{3}=xv_{x}+yv_{y}$ is not considered in (41) due to its severe nonlinearity. It is difficult to obtain a closed-form solution with $u_{3}=xv_{x}+yv_{y}$.

**Figure 7:**
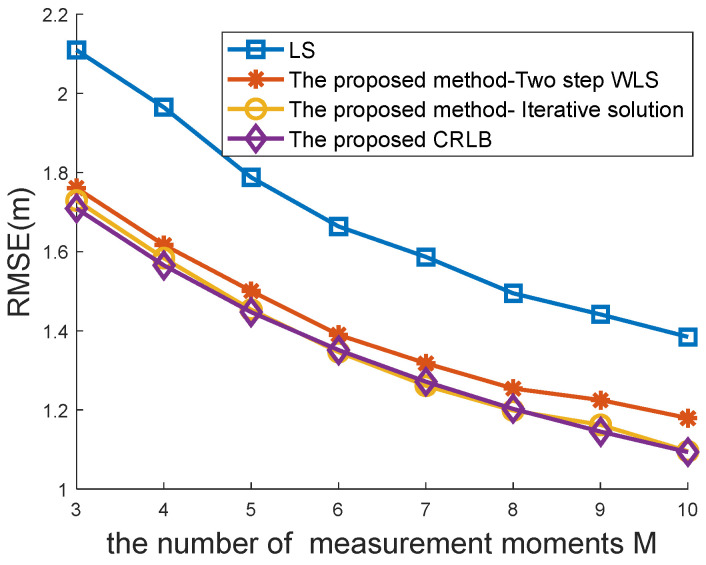
Comparison of the proposed methods under different Ms.

Performance comparisons with different Ms and σ=2m are recorded in Figure 7. This simulation also proved the effectiveness of the proposed methods with closed-form and iterative estimators.

**Figure 8:**
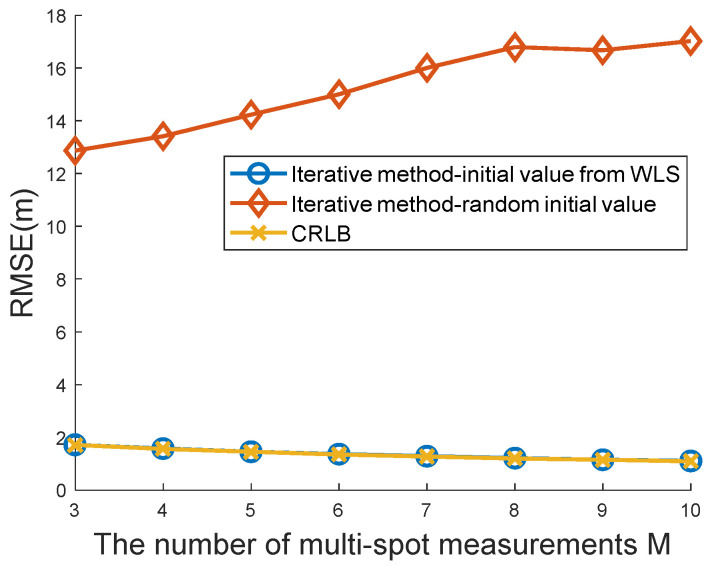
Comparison of the proposed iterative methods with different initial values.

An inappropriate choice of the initial value will lead to divergence in the iterative method. Figure 8 shows the effects of the initial value on the iterative solution. Both kinds of initial values were used in the simulations. The first kind of initial value was calculated from a WLS closed-form solution (48), whereas the other was generated by a uniform random number. It can be seen from Figure 8 that a random initial value will result in a large positioning error and a good initial guess from the WLS estimator (48) to always keep the iterative method convergent to the global minimum. This implies that hybrid architecture containing the proposed WLS estimator and iterative method may obtain a higher positioning accuracy and more stable localization.

## 7. Conclusions

This paper proposes two novel TOA localization methods with closed-form and iterative solutions for a multi-spot measurement model. Compared with the existing location method based on one-spot measurements, multi-spot measurements and motion information are utilized in the proposed method to reduce location errors. The CRLB for the proposed method was derived. The relationship between the proposed CRLB and traditional CRLB was also investigated. The simulation results show that the proposed algorithms have a higher positioning accuracy than the traditional methods. In addition, the proposed iterative method achieved optimal performance since it could attain the corresponding CRLB. A summary table comparing the proposed and traditional methods is provided in Table 1.

## Figures and Tables

**Table 1:** Comparison between the traditional and proposed location methods.

	Traditional Location Methods	Proposed Location Methods
Model	Single-spot measurement model	Multi-spot measurement model
CRLB	$tr\left\{{\left({\left(\frac{H_{1}H_{1}^{T}}{\sigma^{2}}\right)}^{-1}\right)}_{{}_{2\times 2}}\right\}$	$tr\left\{{\left({\left(\frac{\sum_{j=1}^{M}H_{j}H_{j}^{T}}{\sigma^{2}}\right)}^{-1}\right)}_{{}_{2\times 2}}\right\}$
The unknown parameters to be estimated	$\left(x,y\right)$	$\left(x,y,v_{x},v_{y}\right)$
The number of measurement equations	N	N*M
Positioning accuracy	Low	High

## Data Availability

Not applicable.
